# Immunogenic profiling of metastatic uveal melanoma discerns a potential signature related to prognosis

**DOI:** 10.1007/s00432-023-05542-z

**Published:** 2024-01-21

**Authors:** Jian Wang, Miaomiao Liu, Jiaxing Sun, Zifeng Zhang

**Affiliations:** 1 Department of Neurosurgery, Tangdu Hospital, Fourth Military Medical University, Xi’an, China; 2 Department of Respiratory, Tangdu Hospital, Fourth Military Medical University, Xi’an, China; 3 Department of Ophthalmology, Eye Institute of Chinese PLA, Xijing Hospital, Fourth Military Medical University, Xi’an, 710032 China

**Keywords:** Uveal melanoma, TCGA, GEO, Prognosis, Weighted gene co-expression network analysis, Tumor microenvironment

## Abstract

**Background:**

Uveal melanoma (UM) is an aggressive intraocular malignant tumor. The present study aimed to identify the key genes associated with UM metastasis and established a gene signature to analyze the relationship between the signature and prognosis and immune cell infiltration. Later, a predictive model combined with clinical variables was developed and validated.

**Methods:**

Two UM gene expression profile chip datasets were downloaded from TCGA and GEO databases. Immune-related genes (IRGs) were obtained from IMPORT database. First, these mRNAs were intersected with IRGs, and weighted gene co-expression network analysis (WGCNA) was used to identify the co-expression of genes primarily associated with metastasis of UM. Univariate Cox regression analysis screened the genes related to prognosis. LASSO-Cox established a risk score to distinguish high-risk group and low-risk group. Then the GSEA enrichment pathway and immune cell infiltration of the two groups were compared. And combined with clinical variables, a predictive model was constructed. The time-dependent receiver operating characteristic (ROC) curve, calibration curve, and decision curve analysis (DCA) curve were used to verify the stability and accuracy of the final predictive model, and a nomogram was then drawn.

**Results:**

The MEblack, MEpurple, and MEblue modules were significantly associated with the metastasis of UM patients (*P* value < 0.001, = 0.001, = 0.022, respectively). Four genes (UBXN2B, OTUD3, KAT8, LAMTOR2) were obtained by Pearson correlation analysis, weighted gene correlation network analysis (WGCNA), univariate Cox, and LASSO-Cox. And a novel prognostic risk score was established. Immune-related prognostic signature can well classify UM patients into high-risk and low-risk groups. Kaplan–Meier curve showed that the OS of high-risk patients was worse than that of low-risk patients. In addition, the risk score played an important role in evaluating the signaling pathway and immune cell infiltration of UM patients in high-risk and low-risk groups. Both the training set and validation set of the model showed good predictive accuracy in the degree of differentiation and calibration (e.g., 1-year overall survival: AUC = 0.930 (0.857–1.003)). Finally, a nomogram was established to serve in clinical practice.

**Significance:**

UM key gene signature and prognosis predictive model might provide insights for further investigation of the pathogenesis and development of UM at the molecular level, and provide theoretical basis for determining new prognostic markers of UM and immunotherapy.

**Supplementary Information:**

The online version contains supplementary material available at 10.1007/s00432-023-05542-z.

## Introduction

As the most common primary intraocular malignancy among adults (Jager et al. 2020), cancer cells of uveal melanoma (UM) originate from melanocytes within the uveal tract of the eye. In the United States, the mean age-adjusted incidence attains 5.1 per million (Singh et al. 2011). About 85% of the tumor cases derive from the choroid whereas the remaining cases derive from the iris (3–5%) and ciliary body (5–8%) (Damato 2012). The early symptoms of UM are characterized by blurred vision, central dark spots, eye pain, and scleral congestion. Some certain advances have been made in the treatment of UM (Kashyap et al. 2016*).* As suggested by the National Comprehensive Cancer Network (NCCN) guideline, clinically, UM is usually treated by the combination of enucleation, local resection, stereotactic radiotherapy, brachytherapy, and phototherapy (Rao et al. 2020). However, over the past decades, the mortality rate is still rather high (Spagnolo et al. 2016). Metastasis approximately occurs in 50% of patients diagnosed with primary UM (Kujala et al. 2003), with the liver being the most common area involved. Once metastasis occurs, patients with metastatic UM manifest a median overall survival (OS) of less than 12 months (Patel 2013). Further, the response rates of monotherapy were consistently in the single-digit percentage range in a panel of previous studies while the combined immune checkpoint blockade (ICB) revealed higher response rates and better survival outcomes, albeit at the cost of high immune-related toxicity (Heppt et al. 2017a, 2017b, 2019; Kottschade et al. 2016; Algazi et al. 2016; Zimmer et al. 2015). Tebentafusp is a novel treatment modality and the only approved systemic therapy for uveal melanoma (Balushi et al. 2023). Notably, Tebentafusp achieved the best results compared to combined ICB and other systemic treatments; admittedly, the efficacy of this drug needs to be confirmed in more phase 3 clinical trials (Petzold et al. 2023).

To date, there are few studies on the co-gene expression patterns of UM (Kashyap et al. 2016; Zhang et al. 2014), which may limit the exploration of hub genes associated with the prognosis of the disease.

At present, immunotherapies including ICB, cancer vaccines, and chimeric antigen receptor T cell immunotherapy (CAR-T) have become a novel clinical strategy for treating various malignant tumors (Igarashi and Sasada 2020). Nevertheless, the overall therapeutic effect of immunotherapy for UM patients remains not satisfactory (Fu et al. 2022). The adverse response of UM to immunotherapy highlights the lack of knowledge about how metastatic UM develops immune resistance or evades immune surveillance. Interestingly, previous studies have identified that the special anatomic site of UM may contribute to its distinctive immune resistance (Bronkhorst and Jager 2012; Niederkorn 2012). As an immune-privileged organ, the eye serves to restrict the amount of inflammation caused by immune to occur in this region. To be specific, the eye aqueous humor contains a large number of anti-inflammatory and immunosuppressive cytokines, including transforming growth factor-β (TGF-β) and macrophage migration inhibitory factor (MIF) (Niederkorn 2012). Thus, it is urgent to better figure out the physiologic immunosuppressive microenvironment to improve individualized treatment for patients with UM.

In the present study, we sought to start with characterizing the immunophenotype of UM, and then identify the gene modules associated with metastasis using weighted gene correlation network analysis (WGCNA) analysis. Key genes were further identified by univariate Cox, least absolute shrinkage and selection operator (LASSO)-Cox analysis to construct a risk score. Moreover, CIBERSORT analysis was used to evaluate and quantify the degree of immune cell infiltration among low- and high-risk UM patients, and then a predictive model was constructed by combining clinical variables.

## Materials and methods

### Dataset collection

mRNA expression profiles and the corresponding clinical characteristics of UM samples were extracted from Gene Expression Omnibus (GEO) database (https://www.ncbi.nlm.nih.gov/gds) including GSE22138. Based on the GPL570 platform, this dataset included 63 UM samples. The array probe name was converted to a matching gene name according to the platform annotation information. In addition, the mRNA expression profiles along with the clinical traits of 80 UM samples were downloaded from The Cancer Genome Atlas (TCGA) database (https://portal.gdc.cancer.gov/). We used the GSE22138 dataset as the training set whereas we utilized the TCGA-UM datasets as the testing sets. The expression data of genes in the training set and testing sets were standardized through the “limma” package. After merging, R software “sva” (Xie et al. 2021a) package was used to eliminate batch effect.

### The collection of immune-related mRNA

According to the IMPORT database (https://www.immport.org/resources) list of immune-related genes (IRGs), IRGs were screened. The expression of annotated genes in GSE22138 was crossed with the expression of IRGs (Pearson correlation coefficient was greater than 0.3, and *P* value was less than 0.001). Ultimately, 23,516 immune-related mRNAs were obtained.

### Construction of the co‑expression modules

Using the WGCNA package of R software (Langfelder and Horvath 2008), Pearson correlation coefficients between genes were calculated, and appropriate soft threshold β was selected to construct scale-free networks. The optimal soft threshold for adjacency calculation was determined using graphical methods. The topological overlap matrix (TOM) was then constructed from the adjacency matrix. The genes with similar expression patterns were divided into a class and constituted a gene co-expression module. The minimum number of genes in the co-expression modules was set as 50 for the high reliability of the results. Based on TOM matrix, the average linkage hierarchical clustering method was used to cluster genes, and the minimum module was set according to the criteria of hybrid dynamic cutting tree. A weighted co-expression network was constructed to screen hub genes (Chen et al. 2019). The clinical traits of patients in this study included age, sex, presence or absence of metastasis, and time of death. The gene significance obtained in WGCNA meant the correlation between a gene and a clinical trait whereas high gene significance meant this gene was highly correlated with the clinical trait.

### Construction and verification of the risk score signature

The univariate Cox regression analysis was performed to screen out the potential hub genes. The survival-related genes (*P* < 0.05) were then enrolled into the subsequent LASSO analysis. The minimum value of the partial likelihood deviance represented the optimal performance of the model. Consequently, the lambda value with the lowest corresponding deviance was chosen, and this algorithm will output a best model with minimum number of genes. The penalty parameter lambda was detected using tenfold cross-validation (Friedman et al. 2010). Furthermore, independence analysis of the signature was conducted through multivariate Cox regression analyses, and *P* < 0.05 was taken as statistically significant. Four genes with prognostic significance were screened, and the risk score was constructed using the four genes. The risk score was calculated using the following formula:

$${\text{Risk score }}\left( {{\text{patients}}} \right){ } = { }\mathop \sum \limits_{i = 1}^{n} {\text{Coefi * Expi}}$$

In this formula, Coefi is the coefficient and Expi is the expression value of immune-related mRNA. By the survminer R package (Ramos et al. 2017), we calculated an optimal cut-off value for best division of low- and high-risk groups who differed in their survival time both in the training set and validation set. Kaplan–Meier survival analysis was used to determine the difference in OS between the two groups.

### Four gene expression level analysis

To further characterize the four genes used to construct the risk score, we performed gene expression profiling. Cancer Cell Line Encyclopedia (CCLE) (https://sites.broadinstitute.org/ccle) database was applied to obtain mRNA expression levels of four genes in cell lines (Nusinow et al. 2020). We further investigated gene expression in 33 types of cancer and normal tissues from TCGA datasets using the enhanced version of tumor immune estimation resource (TIMER2.0) (http://timer.cistrome.org/) (Li et al. 2020).

### Gene set enrichment analysis (GSEA) enrichment analysis

The single-sample GSEA (ssGSEA) analysis was performed to identify differences in the set of genes expression between the high-risk and low-risk groups. Subsequently, we performed ssGSEA (Subramanian et al. 2005) to obtain further insights into the differences in enrichment pathways between high-risk and low-risk groups. In ssGSEA analysis, four R packages “limma”, “GSVA”, “GSEABase”, “ggpubr”, and “shape2” were used.

### Analysis of immune cell infiltration

To predict the proportion of infiltrating immune cells in tumor tissue, the CIBERSORT bioinformatics computational tool was used. Deconvolution algorithm was used to calculate the infiltration of different types of immune cells between high-risk and low-risk groups. The “corrplot” package was used to deconvolute the correlation between 22 types of tumor-infiltrating immune cells in UM.

### Construction and verification of prognostic-related predictive model

The predictive model was constructed based on age, gender, and risk score. In addition, we used time-dependent receiver operating characteristic (ROC), calibration curve, and decision curve analysis (DCA) curve analysis to assess the predictive value of this signature. To facilitate the prediction of 1-, 3-, and 5-year OS of UM patients, we developed nomogram using the “survival” and “regplot” R packages.

### Statistical analysis

All statistical *P* values were two sided, and *P* < 0.05 was considered statistically significant. All data analysis was performed in R 4.0.1 software (64-bit; https://www.r-project.org/).

## Results

### Extraction and screening of immune-related mRNAs

**Fig. 1:**
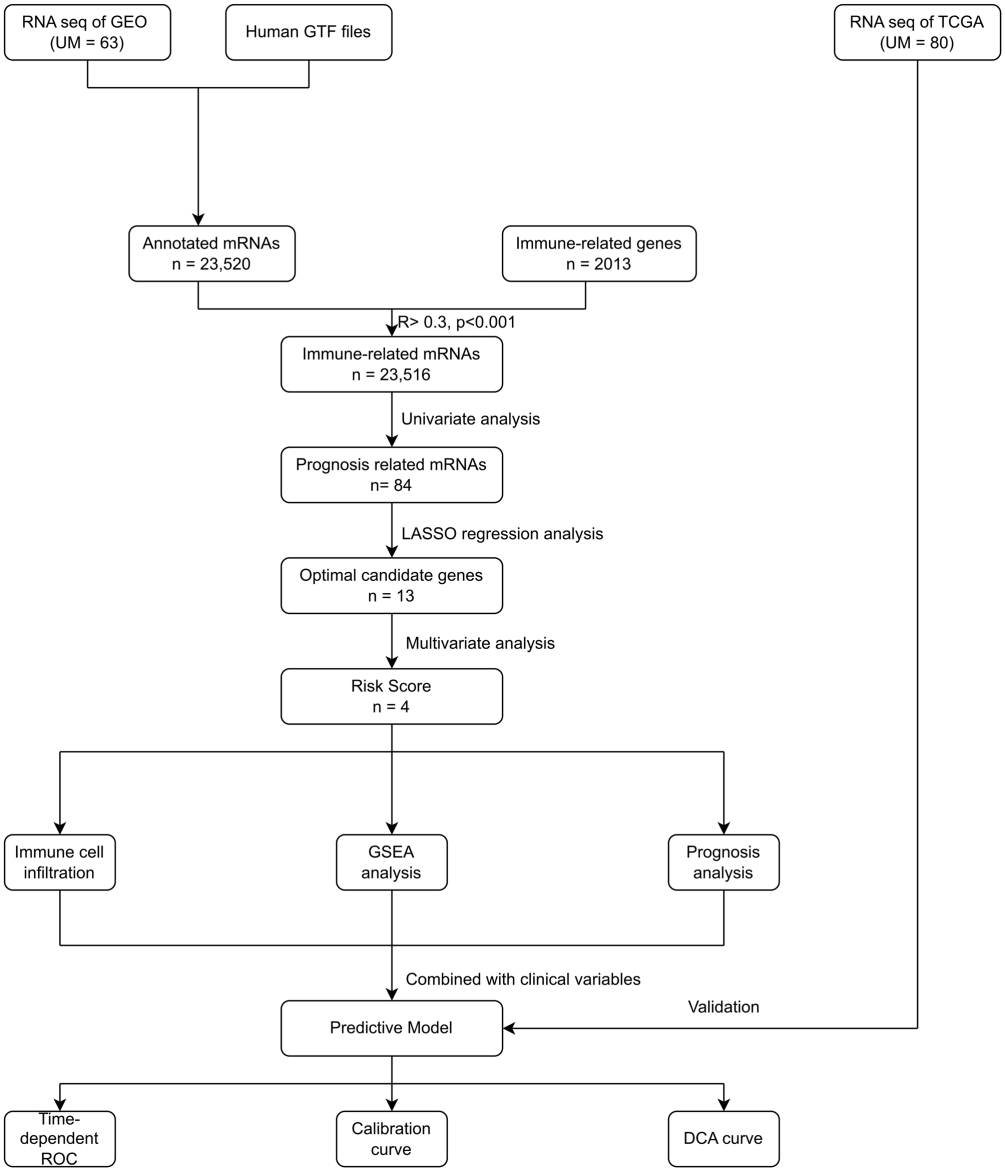
Flow chat of the study. *GEO* gene expression omnibus; *GTF* gene transfer format; *UM* uveal melanoma; *TCGA* The Cancer Genome Atlas; *LASSO* least absolute shrinkage and selection operator; *GSEA* gene set enrichment analysis; *ROC* receiver operating characteristic; *DCA* decision curve analysis

The process of data extraction and processing is shown in Fig. 1. First, we obtained expression profile data and corresponding clinical information of 63 tumor samples from GSE22138. Human GPL570 platform was used to annotate genes and obtain 23,520 mRNA expression data. Subsequently, 2013 IRGs were obtained from the IMPORT database. By Pearson correlation analysis (Pearson correlation coefficient greater than 0.3, *P* value less than 0.001), a total of 23,516 immune-associated mRNAs were identified for subsequent analysis.

### Construction of the co‑expression modules

**Fig. 2:**
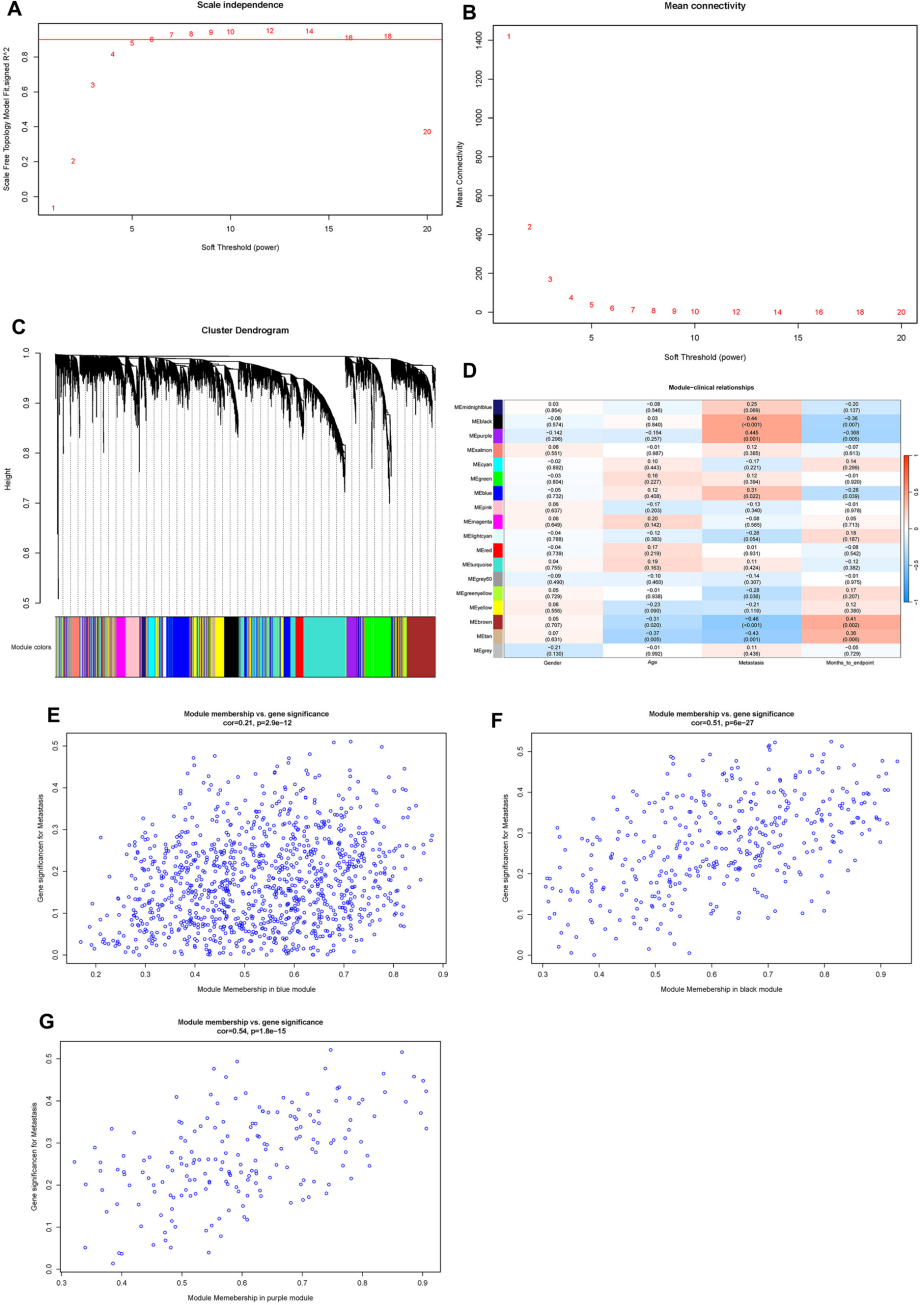
Construction of the co-expression modules via WGCNA in the training set. *WGCNA* weighted gene co-expression network analysis; (cor), correlation. **A** Soft threshold β screening process 1; **B** Soft threshold β screening process 2; **C** Clustering tree of genes (a total of 18 coexpressed gene modules are obtained, which are represented by different colors at the bottom); **D** Heat map of correlation between modules and clinical traits (horizontal axis represents clinical traits, different colors on the left vertical axis represent different modules, each grid marks the correlation coefficient, and the corresponding P-value is in parentheses. The darker the color, the greater the degree of correlation); **E** Scatter plots of gene and model correlation and gene and trait correlation (blue module); **F** Scatter plots of gene and model correlation and gene and trait correlation (black modules); **G** Scatter plots of gene-model correlation and gene-trait correlation (purple module)

The first step of WGCNA analysis is to calculate the soft threshold β. It can be seen from Fig. 2 that when the soft threshold reaches 5, the curve tends to be gentle, and the constructed network is more in line with the scale-free network characteristic (Fig. 2A, B). The 18 co-expressed gene modules were obtained by WGCNA analysis. Based on the module genes, the module cluster tree was constructed. The cluster tree of genes showed the module division process (Fig. 2C). Pearson correlation coefficient and *P* value between each module and clinical traits were calculated (Fig. 2D). In this paper, the coefficient threshold of the module-clinical trait relationship was set at 0.3 ((> 0.3 means positively related to metastasis and <  − 0.3 means negatively associated with metastasis)). As shown in the figure, the Black, Purple, and Blue modules were significantly positively correlated with metastasis (*r* = 0.44, *P* < 0.001; *r* = 0.445, *P* = 0.001; *r* = 0.31, *P* = 0.022), and the genes contained in these three modules were included in the next analysis (Fig. 2E–G).

### Prognostic gene screening and construction of risk score

**Fig. 3:**
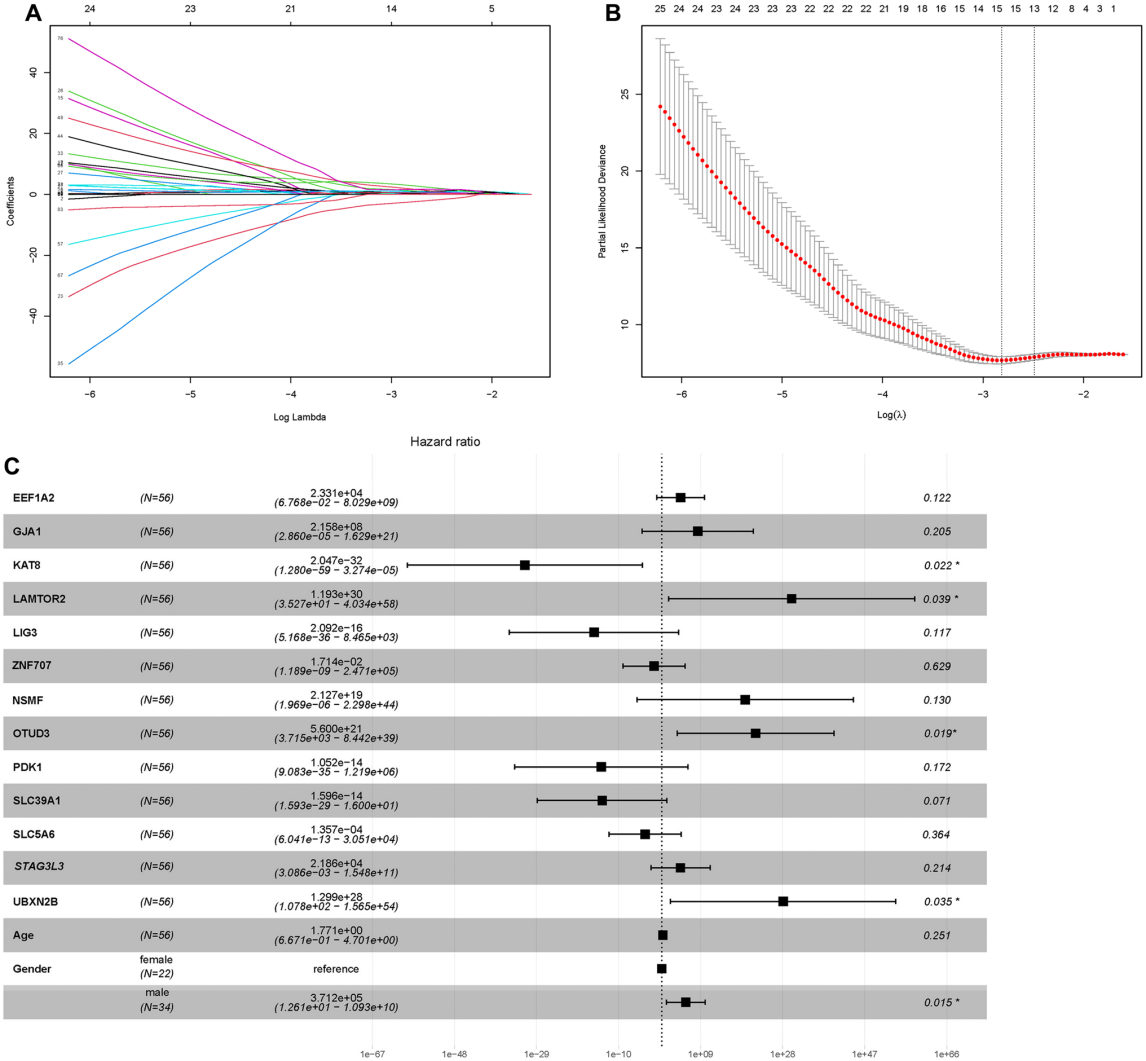
The screening out of gene-based risk score signature for patients with UM in the training set. *UM* uveal melanoma

First, univariate Cox analysis was performed to obtain 82 prognostic mRNAs (*P* < 0.05). Then the LASSO regression analysis was iterated 1000 times, the data features were reduced in dimension, and 13 optimal candidate genes were obtained (Fig. 3A, B), (for example, EEF1A2, GJA1, KAT8, LAMTOR2, LIG3, ZNF707, NSMF, OTUD3, PDK1, SLC39A1, SLC5A6, STAG3L3, UBXN2B, respectively.). The forest map showed the relationship between the 13 selected candidate genes and prognosis (Fig. 3C). Then multivariate Cox analysis was performed, and finally four genes (UBXN2B, OTUD3, KAT8, LAMTOR2) were included to establish the risk score.

**Fig. 4:**
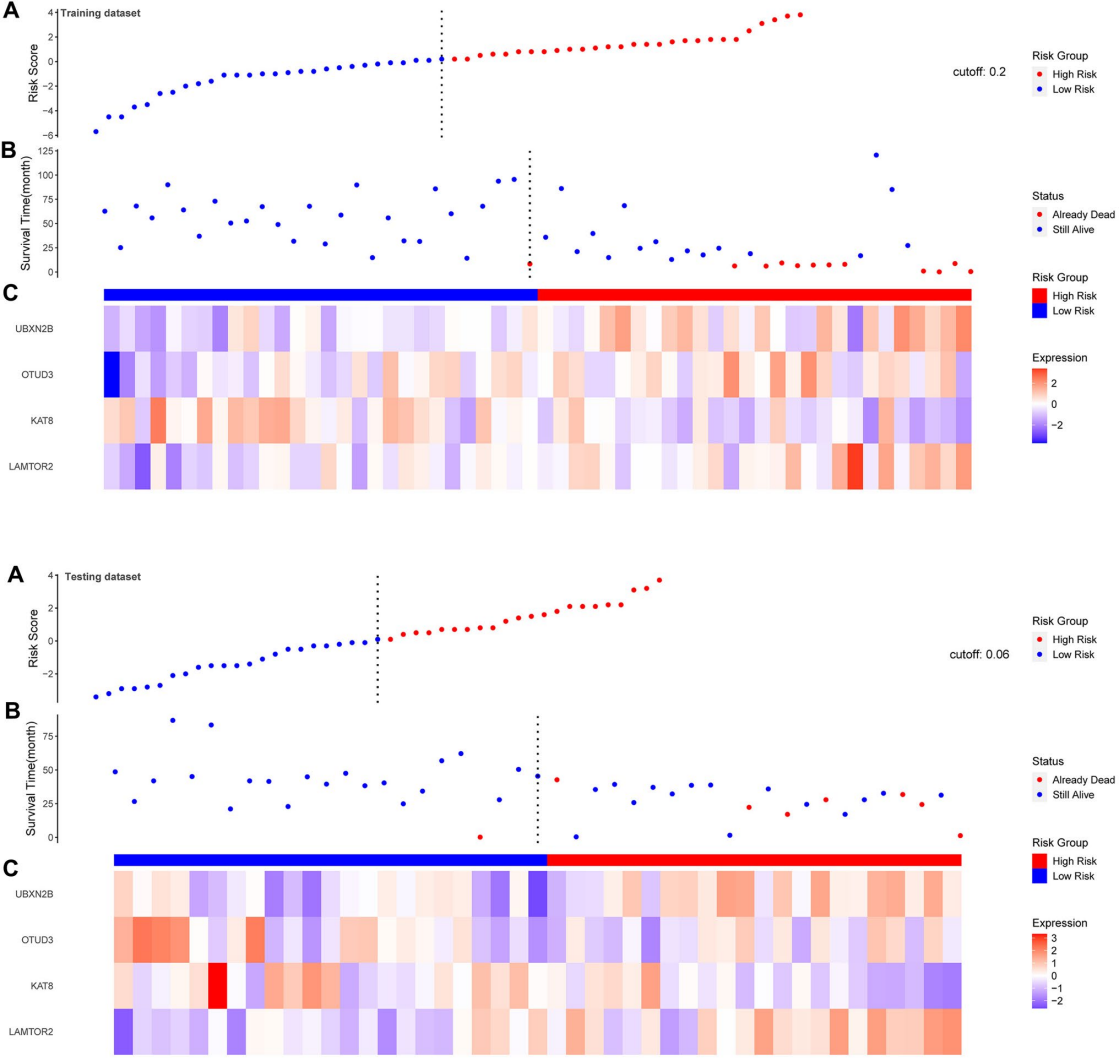
The survival status of the patients in high-risk and low-risk groups

**Fig. 5:**
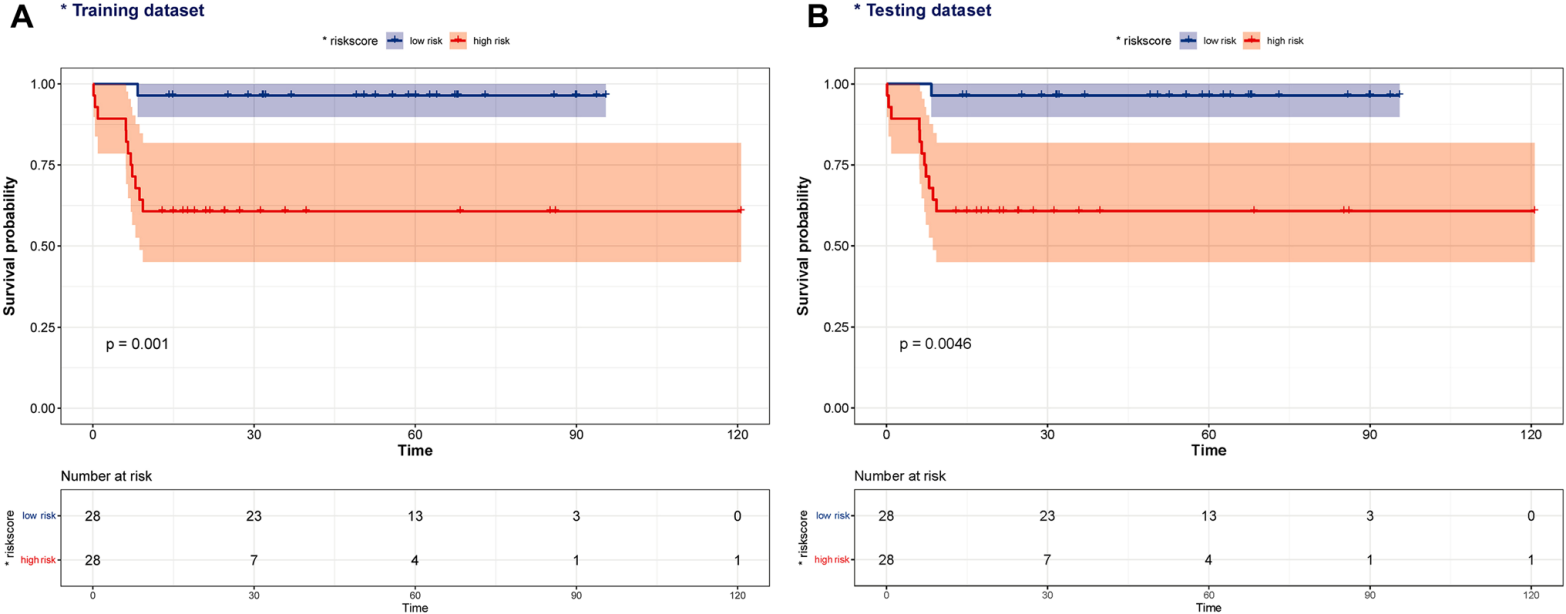
Survival analysis

After construction of the four-gene-based OS risk score signature, UM patients in the training set (GSE22138) were divided into low- and high-risk groups using the risk scores calculated by the optimal cut-off value. As shown in the figure, with the increase of risk score, the survival time presented a trend of shortening. Moreover, the proportion of death in the high-risk group was higher than that in the low-risk group (Fig. 4A, B). This may indicate that our gene risk score could nicely predict the progression and aggressiveness of UM. The Kaplan–Meier (KM) curve demonstrated that in both the training set and the validation set, high-risk patients had significantly poorer OS than low-risk ones (log-rank *P* < 0.05) (Fig. 5A, B). Subsequently, we performed the forest plot to evaluate the prognostic value of the risk score calculated by the formula mentioned above. And we found that the risk score was a prognostic factor for UM in both the training and testing sets (training set: hazard ratio (HR) = 1.11 (1.05, 1.06), *P* < 0.001; testing set: HR = 1.12 (1.05, 1.19), *P* < 0.001) (Supplementary Fig. 1).

### Analysis of gene expression profile

First, we examined the mRNA expression level of these four genes (UBXN2B, OTUD3, KAT8, LAMTOR2) in CCLE UVM cell lines. For example, we found that the mRNA expression level of UBXN2B was highest in WM3772F, followed by MEL270 and 921. In terms of OTUD3, the mRNA expression level was highest in 921, followed by MEL202 and MEL270. Details can be seen in Supplementary Fig. 2. Taking TCGA data, compared with adjacent normal tissues, the UBXN2B expression level was significantly upregulated in breast invasive carcinoma (BRCA), cholangiocarcinoma (CHOL), esophageal carcinoma (ESCA), head and neck squamous cell carcinoma (HNSC), head and neck squamous cell carcinoma (HNSC)-human papilloma virus (HPV), liver hepatocellular carcinoma (LIHC), and stomach adenocarcinoma (STAD), but downregulated in kidney chromophobe (KICH), kidney renal clear cell carcinoma (KIRC), kidney renal papillary cell carcinoma (KIRP), lung squamous cell carcinoma (LUSC), skin cutaneous melanoma (SKCM), and thyroid carcinoma (THCA). Details can be seen in Supplementary Fig. 3.

### GSEA enrichment analysis

**Fig. 6:**
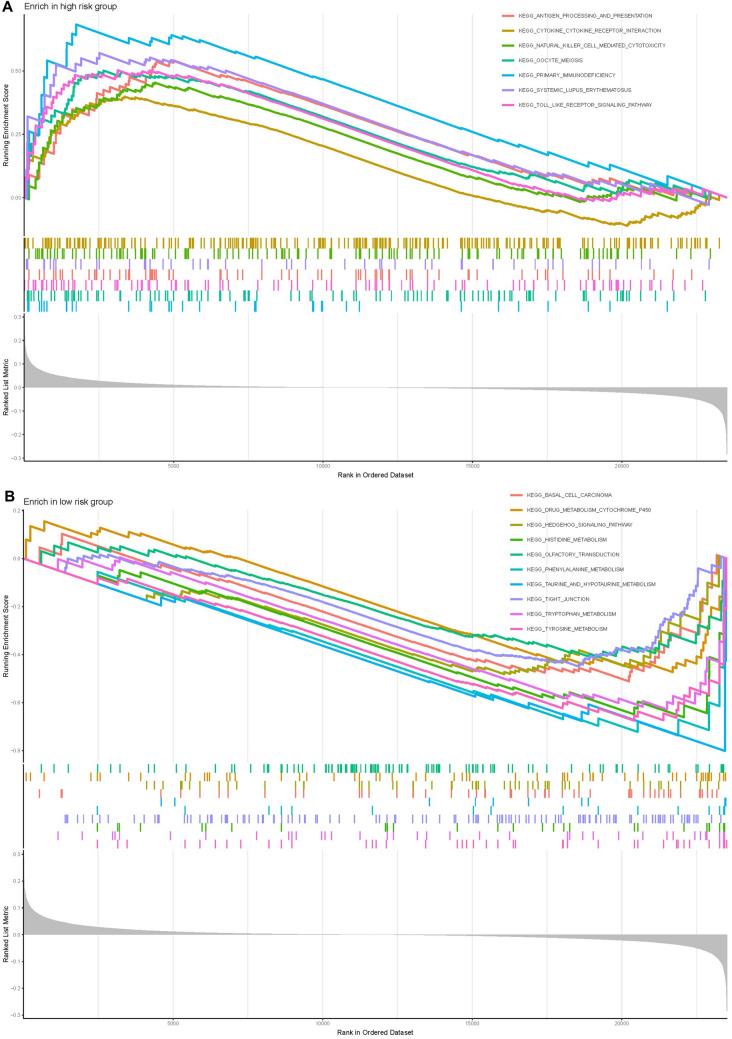
GSEA based on the immune-related mRNAs risk model. *GSEA* gene set enrichment analysis

The results of GSEA enrichment analysis for high-risk and low-risk groups were as follows. The high-risk groups were mainly concentrated in antigen processing and antigen presentation, cytokine receptor interaction, natural killer cytotoxicity, oocyte meiosis, primary immunodeficiency, systemic lupus erythematosus, and toll-like receptor signaling pathways. In contrast, the low-risk group was mainly concentrated in basal cell carcinoma, cytochrome P450 metabolism, Hedgehog signaling pathway, histidine metabolism, olfactory conduction, phenylalanine metabolism, taurine metabolism, tight junction, tryptophan metabolism, and tyrosine metabolism (Fig. 6).

### Analysis of immune cell infiltration

**Fig. 7:**
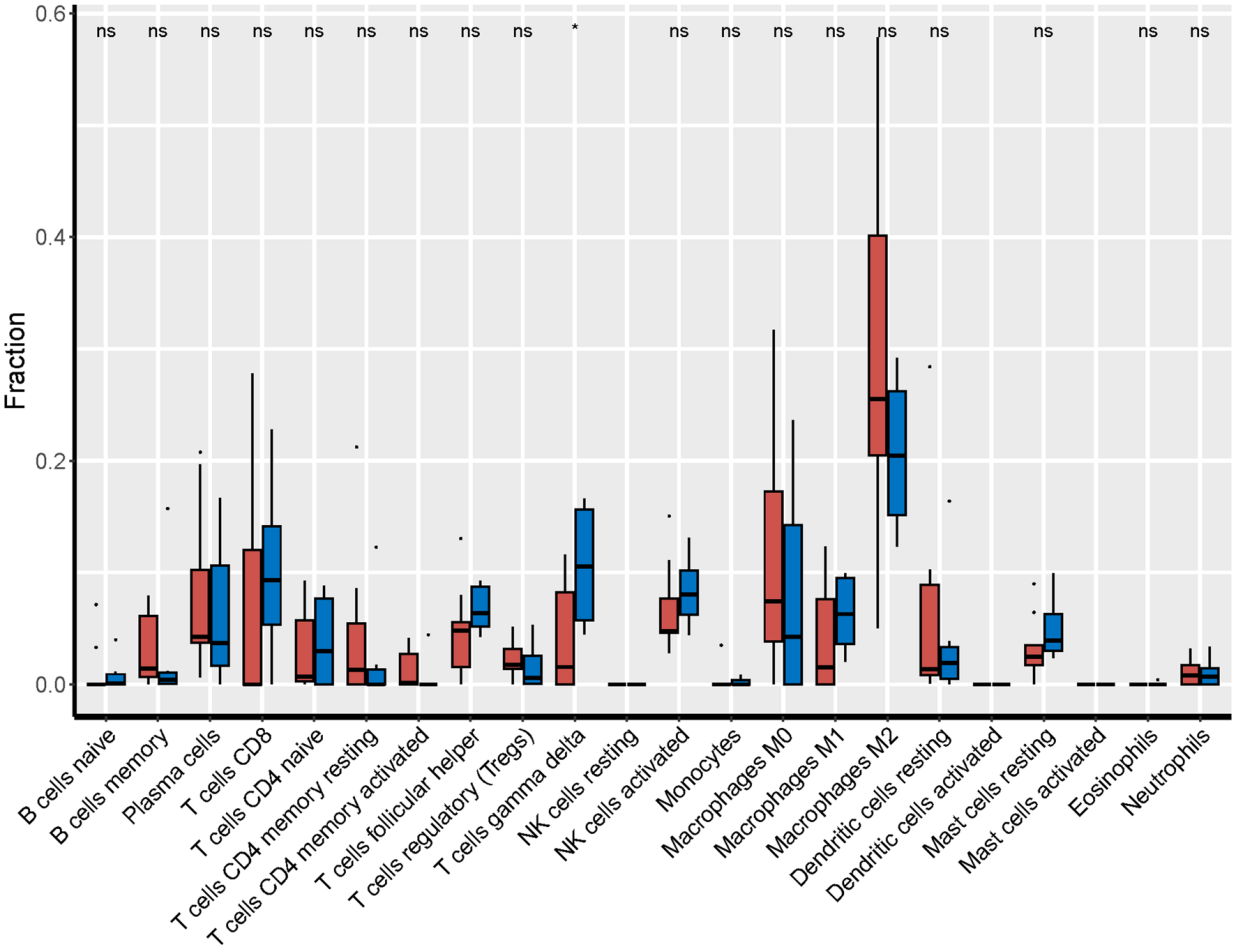
Boxplots of differences in immune cell infiltration between high-risk and low-risk groups

By CIBERSORT analysis, the infiltration of 22 kinds of immune cells in low-risk group and high-risk group was obtained. As shown in the figure, the infiltrating levels of γδT cells were significantly higher in the high-risk group compared with the low-risk group (*P* < 0.05). Furthermore, using the TIMER website (https://cistrome.shinyapps.io/timer/), high γδT cells infiltration was found to be associated with worse OS of UM patients (*P* < 0.05). These results indicated that the bad prognosis might be partly due to the high infiltration of this type of immune cell (Fig. 7).

### Construction of prognostic-related predictive model

**Fig. 8:**
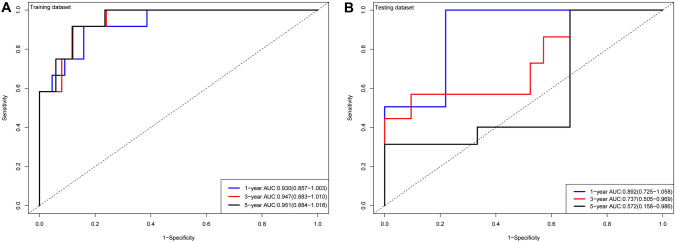
ROC of risk model in the dataset. *ROC* receiver operating characteristic; *AUC* area under the curve

**Fig. 9:**
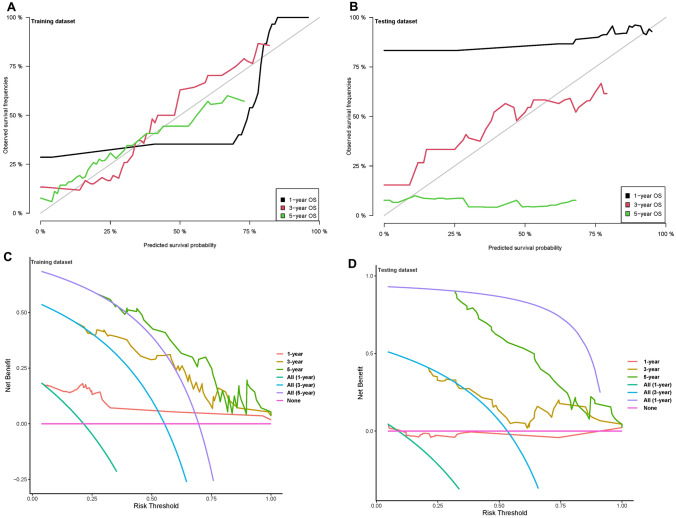
Calibration and DCA curve in the dataset. *DCA* decision curve analysis; *OS* overall survival

**Fig. 10:**
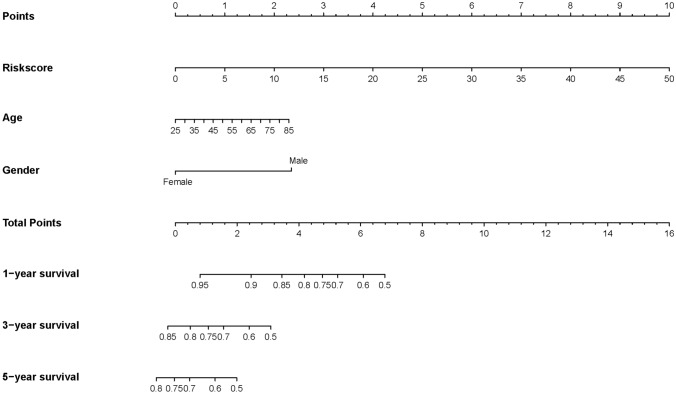
Nomogram was plotted for the prediction of overall survival time

To further verify the clinical significance of risk score, two clinical variables, gender and age, were added for multivariate Cox analysis. In the training set, the survival rates of 1-, 3-, and 5 year were predicted, and the time-dependent area under the curves (AUCs) of the model were above 0.930 (Fig. 8A, B). To be specific, the AUCs of the ROC curve were 0.930 at 1-year, 0.947 at 3-year, and 0.951 at 5-year in the training cohort. In the validation set, the model also showed a good degree of differentiation. The calibration curves of 1 year, 3 years, and 5 years in the training set were close to the diagonal line, which indicated that the model had a good calibration degree (Fig. 9A, B). However, the performance of calibration curve in the validation set was not as good as that of the verification set, which may be due to the fact that the sample size was not large enough and thus, the classifier was weak. In the training set, the 1-, 3- and 5-year DCA curves were all between None and ALL, showing a good net benefit, whereas in the verification set, the 1-year and 3-year DCA curves performed better (Fig. 9C, D). To facilitate clinical use, this paper further constructed a nomogram composed of age, gender, and risk score. After calculating the score of each variable and combining the total score, the survival rate of 1 year, 3 years, and 5 years can be calculated (Fig. 10).

## Discussion

Despite its low incidence, UM is still the most common primary intraocular malignant tumor in adults, and is characterized by its high mortality (> 95%), high metastasis (> 50%), and poor prognosis, making the exploration for effective biomarkers for assessing prognosis crucial (Marseglia et al. 2021). In this study, WGCNA were used to screen out metastasis-related co-expression modules from the GEO database. The Black, Purple, and Blue modules were significantly associated with the progression and metastasis in UM. Then we used univariate Cox, LASSO-Cox regression to screen out four genes associated with prognosis. The GSEA analysis demonstrated that multiple immune-related pathways were upregulated in the high-risk group. Subsequently, we used these four prognosis-related genes to construct a risk score signature, which was found to be an independent factor for predicting OS. Moreover, combined with other age, gender, a clinical predictive model was established. Both the survival curves and ROC analysis results showed the robustness and reliability of the predictive model for prognosis prediction of UM patients. The AUCs of the ROC curve in the training cohort for 1-year, 3-year, and 5-year were 0.930, 0.947, and 0.951, respectively. This predictive model helps simplify clinical individualized monitoring and treatment strategies for UM patients.

Four genes included in the predictive model have not been adequately studied in UM. As indicated by previous study, downregulation of OTUD3 was associated with poor prognosis in patients with esophageal cancer. The specific mechanism may be that OTUD3 directly interacts with zinc finger protein ZFP36, and stabilizes ZFP36 by inhibiting FBXW7-mediated polyubiquitination of K48 junction (Ntunzwenimana et al. 2021). Xie et al. (2021b) found that OTUD3 was significantly overexpressed in hepatocellular carcinoma, and high expression of OTUD3 was correlated with tumor size, distant metastasis, and poor TNM stage. Wang et al. (2021) analyzed the relation between OTUD3 and lymphatic metastasis, and found that nicotine-mediated OTUD3 downregulation inhibited VEGF-C mRNA decay to promote lymphatic metastasis of human esophageal cancer. Qiu et al. (2023) found that acetylation degradation of KAT8 could affect colorectal cancer invasion and migration. Dong et al. (2019) found that MYST1/KAT8/MOF promoted the progression of glioblastoma by activating epidermal growth factor receptor (EGFR) signaling. Previous study also identified KAT8 as a key molecule important for cancer cell survival. To be specific, they found that KAT8 regulated G2/M cell cycle arrest through AKT/ERK-cyclin D1 signaling (Zhang et al. 2013). In addition, LAMTOR3 expression was significantly decreased in renal clear cell carcinoma compared with normal renal tissue. LAMTOR3 may be a potential marker for the diagnosis and treatment of renal clear cell carcinoma (Gong et al. 2022). Song et al. (2015) found that LAMTOR3 polymorphisms may be a potential biomarker of genetic susceptibility to gastric cancer. In a mouse model of pancreatic cancer, the siRNA-FA-PEG-COL nanoparticles against LAMTOR*,* which strongly inhibited retroperitoneal invasion and significantly inhibited peritoneal dissemination compared to the other nanoparticles, improved prognosis of the mice (Taniuchi et al. 2019).

Two clinical variables, age and gender, were also included. Kaliki et al. (2013) found that the older patients with UM had worse prognosis. In addition, they divided UM patients into young adults (< 20 years old), middle-aged (21–60 years old), and senior citizens (> 60), and found that younger people had a lower risk of metastasis compared to middle-aged and older adults. In a survival analysis of 119 patients, Rietschel et al. (2005) found that women had a significantly lower risk of disease-specific death than men. Similarly, a study of 723 patients with UM found that male patients had a worse prognosis compared to female patients (Zloto et al. 2013). Therefore, two variables including age and gender were included in the clinical predictive model for further analysis. Previous studies have tried to reveal the immune infiltration pattern of this special tumor (Luo and Ma 2020; Pan et al. 2020). In this study, we found that γδT infiltration levels were significantly higher in high-risk UM samples than in low-risk ones (*P* < 0.05). Consistent with previous reports (Wang et al. 2020; Lei and Zhang 2021), γδT were identified to be related to poor prognosis in UM patient. Previous studies also have focused on prognosis prediction based on the gene expression profiles of UM. For instance, Vaquero-Garcia et al. (2017) used variables including chromosome characteristics, age, sex, tumor site, and tumor size to predict patients’ prognosis, and the accuracy of model attained 85%. Using TCGA as the training cohort and GSE22138 as the validation cohort, (Luo and Ma 2020) identified 21 prognostic genes related to microenvironment. Xue et al. (2019) conducted univariate and multivariate Cox analysis on the TCGA cohort to establish an 18-gene prognostic model for patients with OS. NDUFB9, NDUFV2, Cyc1, and CTNNB1 were screened out by Choi et al. (2020) as prognostic predictors of UM patients. Luo et al. (2020) performed univariate Cox regression and LASSO-Cox to construct ten gene signatures, and used the GSE22138 cohort to verify the signatures. Compared to Luo et al., the model in our paper is more accurate in predicting 1-year and 3-year OS (AUCs of 0.892 and 0.737, respectively).

Our study had some strengths. First, we identified metastasis-related genes by WGCNA, and then constructed a gene signature based on these genes for better prognosis prediction. Notably, metastasis is significantly associated with the survival of patients. To the best of our knowledge, there is limited literature on the immune signature of metastatic UM tumors (Qin et al. 2017; Rothermel et al. 2016; Krishna et al. 2017; Javed et al. 2017). Second, the model was derived from the GSE22138 cohort and verified by the external data set TCGA cohort. Third, clinical variables including age and gender were added together with risk score to establish a model for predicting UM prognosis.

Admittedly, there are some limitations in our current study. First, these results were obtained from public databases, and therefore need to be validated by experiment. Second, the sample size is not large enough, and further data training of large multi-center samples is needed to improve the performance of the model.

## Conclusion

In conclusion, through WGCNA, our study demonstrated that three co-expression modules were associated with metastasis among UM patients. Through LASSO-Cox analysis, four genes were finally identified for inclusion in the risk score. Furthermore, patients in high-risk group have significantly higher levels of an immune infiltrate (γδT cell) compared with patients in low-risk group. Notably, this model needs to be validated in a larger dataset, and may be conducive to therapeutic decision-making in the clinical setting.

## Supplementary Information

Below is the link to the electronic supplementary material.

Supplementary file1 (DOCX 1139 KB)

## Data Availability

The datasets analyzed during the current study are available in the GEO database (https://www.ncbi.nlm.nih.gov/geo/) (GSE22138) and TCGA database (https://portal.gdc.cancer.gov/) (TCGA-UVM). All data generated or analyzed during this study are included in this published article and its supplementary information files.

## Abbreviations

UM: Uveal melanoma
NCCN: National comprehensive cancer network
OS: Overall survival
ICB: Immune checkpoint blockade
TGF -β: Transforming growth factor-β
MIF: Migration inhibitory factor
WGCNA: Weighted gene correlation network analysis
LASSO: Least absolute shrinkage and selection operator
GEO: Gene expression omnibus
TCGA: The Cancer Genome Atlas
IRGs: Immune-related genes
TOM: Topological overlap matrix
GSEA: Gene set enrichment analysis
ssGSEA: Single-sample GSEA
ROC: Receiver operating characteristic
DCA: Decision curve analysis
KM: Kaplan–Meier
HR: Hazard ratio
AUCs: Area under the curves
EGFR: Epidermal growth factor receptor
