# Supplementary material for: Immunogenic profiling of metastatic uveal melanoma discerns a potential signature related to prognosis
Source: J Cancer Res Clin Oncol. 2024 Jan 21;150(1):23. doi: 10.1007/s00432-023-05542-z (PMC10800307; doi:10.1007/s00432-023-05542-z)
Supplement: Supplementary file 1 — Supplementary file1 (DOCX 1139 KB) [file 432_2023_5542_MOESM1_ESM.docx]

Supplementary figure 1 Forest plots were performed to indicate the hazard ratio of the risk score in predicting patient’ survival (A. Univariate analysis; B. Multivariate analysis).


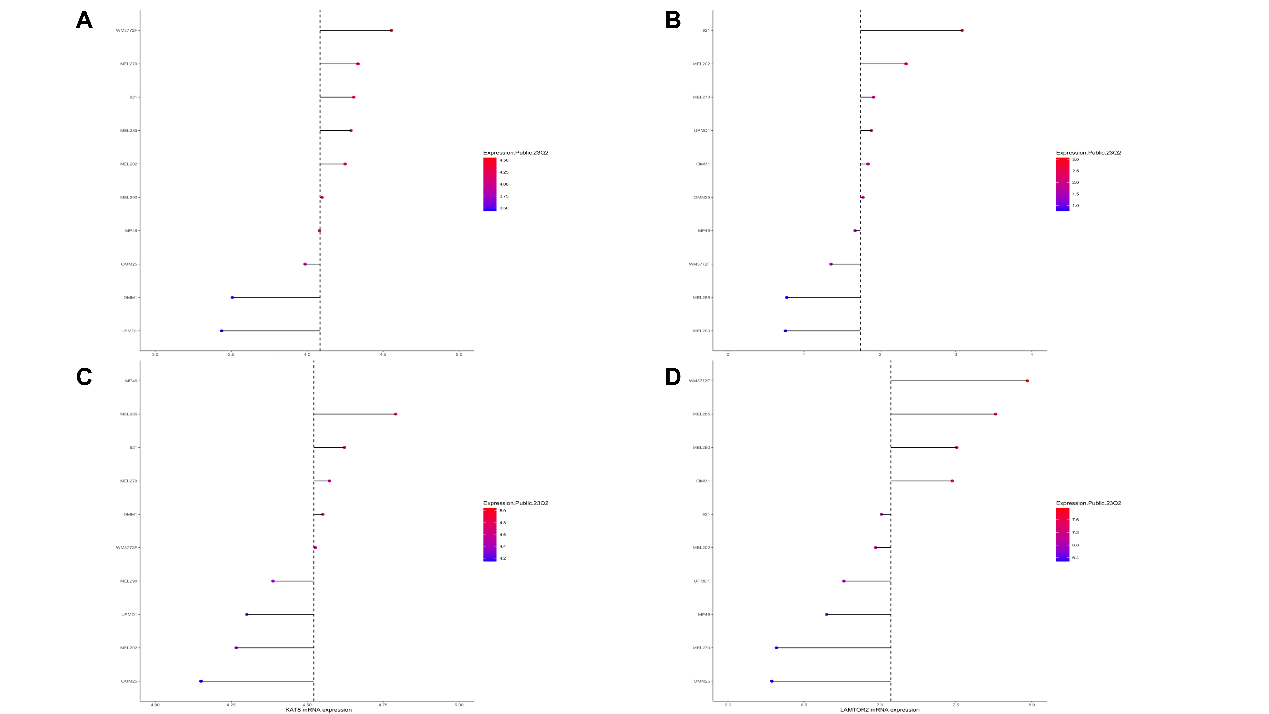


Supplementary figure 2 Gene expression level of four genes in different cell lines of UM.

UM, uveal melanoma


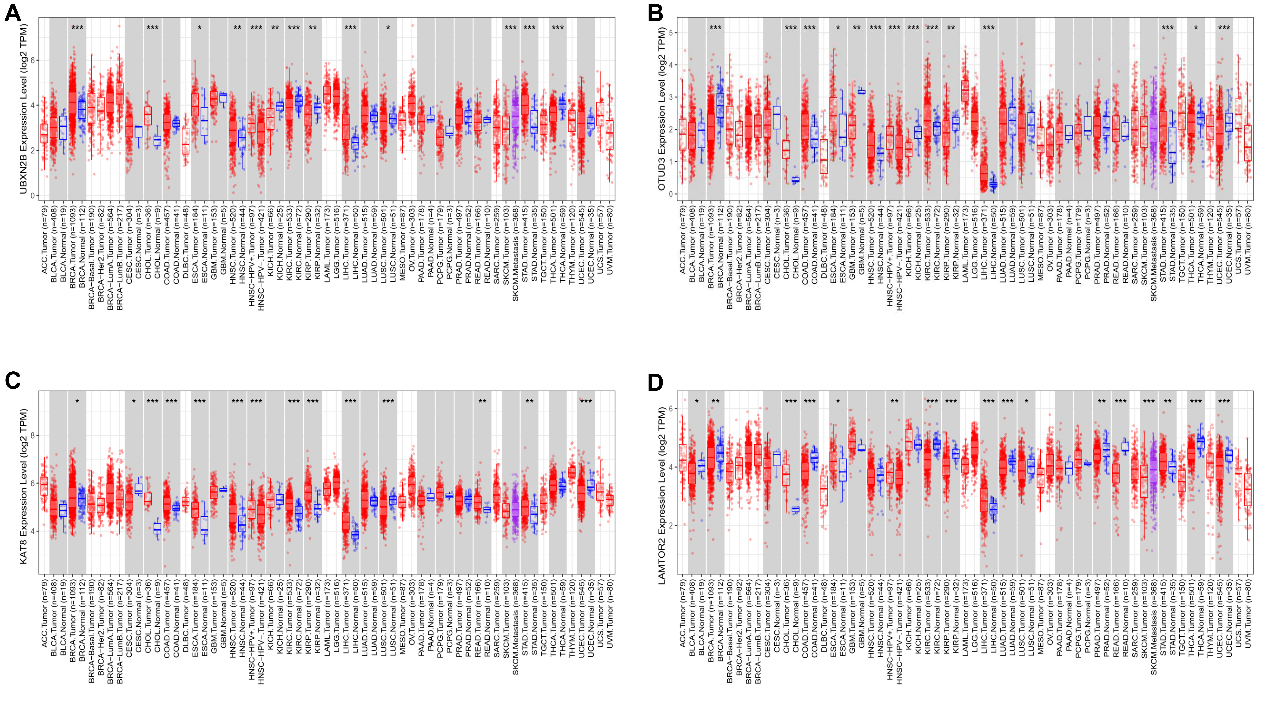


Supplementary figure 3 Gene expression level of four genes in pan-cancer analysis.
